# Patterns of time spent in sedentary behavior, physical activity, and sleep are associated with cognitive decline among CLSA participants: A latent class analysis

**DOI:** 10.1016/j.jnha.2025.100619

**Published:** 2025-06-27

**Authors:** R.A. Palazuelos-González, R.C. Oude-Voshaar, N. Smidt, A.C. Liefbroer

**Affiliations:** a Department of Epidemiology, University of Groningen, University Medical Center Groningen, Groningen, The Netherlands; b Department of Psychiatry, University of Groningen, University Medical Center Groningen, Groningen, The Netherlands; c Netherlands Interdisciplinary Demographic Institute (NIDI)–Royal Netherlands Academy of Sciences (KNAW), Lange Houtstraat 19, The Hague 2511 CV, The Netherlands; d Department of Sociology, Vrije Universiteit Amsterdam (VU), Amsterdam, The Netherlands

**Keywords:** Cognitive decline, CLSA, Latent class analysis, Physical activity, Sedentary behavior, Sleep

## Abstract

**Objectives:**

To identify latent classes of time spent in movement activities (leisure sitting, physical activities, and sleep), characterize them, and assess their association with changes in memory, executive functioning, and overall cognition among middle aged and older adults.

**Design:**

Longitudinal cohort study with a 3-year follow-up.

**Setting:**

Non-institutionalized middle aged and older adults from the general population.

**Participants:**

12,212 adults aged 45–86 years from the Canadian Longitudinal Study on Aging.

**Measurements:**

Latent Class Analysis was performed to identify groups of participants with comparable movement activities based on self-reported time spent in leisure sitting, walking, moderate and vigorous physical activity (assessed using the Physical Activity Scale for the Elderly), and sleep at baseline. Multivariable linear regression models were used to examine the associations between the identified groups and reliable change in memory, executive functioning and overall cognition, measured by a validated neuropsychological battery including 6 different cognitive tests.

**Results:**

Three groups were identified: Sedentary/Disturbed Sleep (SedDS, 53.9%), Intermediately Active/Normal Sleep (IntNS, 34.6%), and Active/Normal Sleep (ActNS, 11.5%). The SedDS group showed greater cognitive decline after 3 year follow-up across all cognitive domains (memory β = −0.061, 95%CI −0.100, −0.021; executive functioning β = −0.049, 95%CI −0.090, −0.008; overall cognition β = −0.067, 95%CI −0.106, −0.027) compared to IntNS. Interestingly, ActNS showed a greater cognitive decline (memory β = −0.065, 95% CI −0.124, −0.005; overall cognition β = −0.062, 95% CI −0.123, −0.002) relative to IntNS.

**Conclusion:**

In mid- and later life, sleep disturbances primarily coincide with a sedentary lifestyle. For optimal cognitive ageing, moderate physical activity seems more beneficial than either sedentary behavior or excessive physical activity to delay accelerated cognitive ageing.

## 1 Introduction

The global burden of dementia is projected to affect over 152 million people by 2050, representing a significant public health challenge [1]. The impact of dementia on individuals, families, and healthcare systems underscores the urgency of reducing its risk or delaying its onset. Among the various modifiable risk factors for dementia and cognitive decline, physical inactivity has been consistently associated with an increased likelihood of both conditions [2]. Despite numerous efforts to promote physical activity, many individuals continue to spend a significant portion of their day in sedentary activities, which have been proposed as contributors of cognitive decline [3]. Sleep, another determinant of brain health, not only supports the neurorestorative process but also interacts in time with physical activity and sedentary behavior, forming a complex web of influences on cognitive function [4]. Although the independent effects of these movement activities on cognition have been previously documented, their combined influence remains unclear.

Evidence indicates that sedentary behavior, physical activity, and sleep each influence cognitive health, although their relative impact varies. Longer durations and higher intensities of physical activity have been associated with a lower risk of dementia and cognitive decline [2,[5], [6], [7]]. In contrast, the role of sedentary behavior is less clear, with some studies linking prolonged sedentary time to an increased risk of mild cognitive impairment [8], while others report no significant association [3]. Additionally, sleep shows a non-linear relationship with cognition, as both insufficient and excessive sleep durations have been identified as potential risk factors for cognitive decline [9]. Notably, insufficient sleep may reduce physical activity levels and increase sedentary time [10], while both physical activity and sedentary behavior can influence sleep duration [11], potentially affecting cognitive health and contributing to the dynamic interplay among these factors. This could imply that the combined influence of these activities on cognitive function may differ from their individual effects. Understanding these interactions not only fills a gap in current research but could lead to more effective interventions aimed at preventing or delaying cognitive decline by targeting multiple behaviors simultaneously.

Given the complex and interdependent nature of these behaviors, traditional analytical methods may not fully capture their combined impact. To address this complexity and variability in daily movement activity patterns, latent class analysis (LCA) could be particularly useful. LCA is a person-centered statistical technique that identifies distinct groups of persons that show comparable behavioral patterns, providing a more detailed view of how various combinations of the movement activities influence cognitive outcomes [12]. Previous studies applying LCA to identify groups based on daily movement activity patterns and their associations with cognitive functioning have been limited to cross-sectional designs, or examined only specific types of physical or sedentary activities [[13], [14], [15], [16]]. Only one included sleep in their analyses [17]. By jointly examining sleep, sedentary behavior and physical activities within LCA models and employing a longitudinal approach, we aim to provide a more comprehensive view of daily movement activities and their association with cognitive decline.

Using data from the Canadian Longitudinal Study on ageing, a cohort designed to understand the complexity of the ageing process in a nationally representative sample with middle age and older adults, this study aims to: 1) identify distinct groups of middle-aged and older people with different movement activity patterns, based on their time spent in sedentary behavior, physical activities (walking, moderate, and vigorous physical activity), and sleep; 2) characterize these groups in terms of their demographic, health, and lifestyle characteristics; and 3) estimate the associations between the identified groups with different movement patterns and changes in cognitive performance over time.

## 2 Methods

### 2.1 Study population

**Fig. 1:**
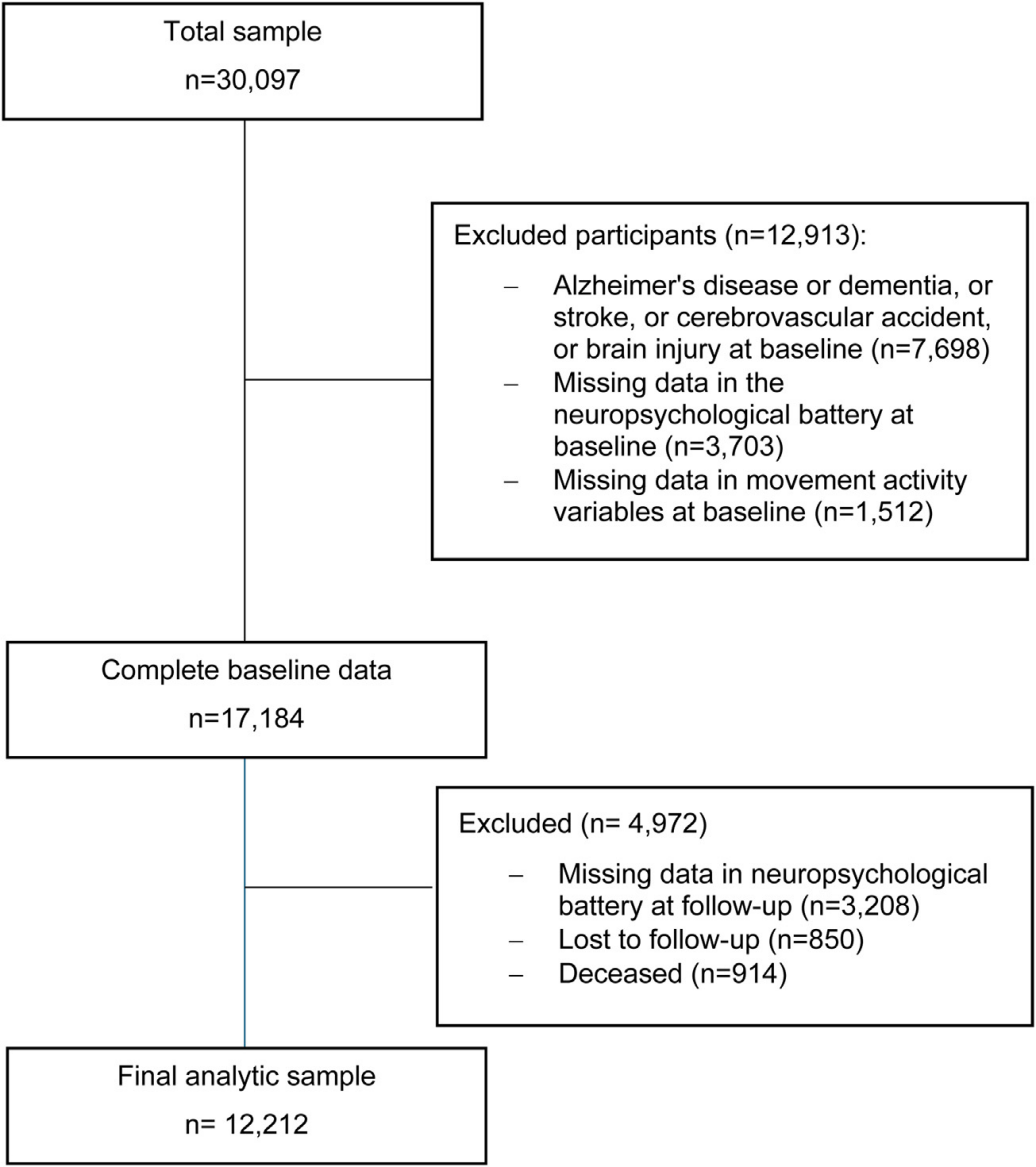
Selection of participants.

The Canadian Longitudinal Study on Aging (CLSA) is a longitudinal cohort study with a nationally representative, stratified, random sample of Canadian adults aged 45–85 years. Participants are assessed every 3 years until 2033 or the participant’s death to gain a better understanding of the ageing process [18]. The study comprises two separate cohorts, the Comprehensive (*n* = 30,097) and the Tracking cohort (*n* = 21,241). For both cohorts the baseline data was collected between 2010 and 2015. The Comprehensive cohort gathered data through both an in-home interview and an in-depth physical assessment conducted at designated collection sites. First follow-up data was collected between 2015 and 2018. The CLSA excluded residents of the Canadian territories and some remote regions, persons on Federal First Nations reserves and other provincial First Nations settlements, full-time members of the Canadian Armed Forces, individuals in institutional settings at recruitment, non-English or French speaking persons, and those not cognitively able (to hear, or answer) to participate on their own at recruitment. Details of the CLSA have been published elsewhere [19]. For this study, we selected the entire sample from the Comprehensive cohort with complete information at baseline and follow-up in the neuropsychological battery as well as in the Physical Activity Scale for the Elderly (PASE) questionnaire at baseline. Exclusion criteria included previous diagnosis of Alzheimer’s Disease or dementia, brain injury, and stroke or ischemic attack. No other exclusion criteria were used except from those applied by the original study. Our final sample consisted of 12,212 participants (Fig. 1).

The CLSA adheres to the Canadian Tri-Council Policy Statement and the Declaration of Helsinki. Ethics approval for the study has been granted by the ethics boards of all 13 participating Canadian universities, with the protocol reviewed annually. Informed consent was obtained from all participants involved in the study.

We tested for initial selection bias by comparing respondents with and without complete baseline data (Appendix A in the Supplementary materials) and for attrition bias by comparing respondents in our final analytic sample to those who dropped out among those with complete baseline data (Appendix B). At both stages, men, those with lower socio-economic status and those with poorer health are more likely to be excluded.

### 2.2 Cognition

Cognition is measured using a neuropsychological test battery including the Rey Auditory Verbal Learning Test (REY I), Verbal Fluency Test (VFT), Mental Alternation Test (MAT) [20], Delayed Rey Auditory Verbal Learning Test (REY II), Stroop Neurological Screening Test (STP) [21,22], and Controlled Oral Word Association Test (COWAT). These tests were chosen based on their psychometric properties, availability in French and English, relevance for the cohort age group, and feasibility in terms of time and costs [23]. Trained interviewers administered the tests face-to-face at baseline and follow-up. Test scores were normed according to age, sex, and education using a neuro-healthy reference sample [24]. Specific cognitive domains were assessed through combinations of tests: memory was evaluated using REY I and REY II, while executive functioning was measured with VFT, MAT, STP, and COWAT. All tests were used to assess overall cognitive performance. Reliable change z-scores, based on baseline and follow-up normed scores, were calculated and used in the analysis of this study [25].

### 2.3 Movement activities

Physical activities and sitting time were assessed using the CLSA-adapted version of the PASE [26]. Participants were asked to report their frequency (never, 1−2 days, 3–4 days, and 5–7 days), and duration (<30 min, 30 min-1 h, 1−2 h, 2−4 h, and ≥4 h) in walking, light, moderate, strenuous, and strength physical activities, during the previous 7 days. Additionally, leisure sitting time was measured by the question “How often did you participate in sitting activities such as reading, watching TV, computer activities or doing handicrafts?”. Response options for frequency and duration were the same as those used for physical activities. We used the midpoints of each frequency and duration to calculate time spent in each activity, except for ≥4 h, where a total duration of 4 h was assigned. This approach has been used previously in other studies [[27], [28], [29]]. Light and moderate activities were combined into light-to-moderate physical activity (MPA), and strength and strenuous activities into vigorous physical activity (VPA). Physical activity variables (walking, MPA, and VPA) were categorized as 0, >0 to <1, and ≥1 h/day. Sitting was categorized in 0 to <2, 2 to <3, and ≥3 h/day.

Total sleep time was assessed with the question “During the past month on average how many hours of actual sleep did you get at night? (This may be different than the number of hours you spent in bed.)” and categorized in <7 h/day, 7−8 hours/day, and >8 h/day [30,31].

### 2.4 Covariates

The following covariates were included based on literature. Age was treated as a continuous variable, while sex was categorized as female (reference) and male. Education was classified as high, middle, or low using the CLSA-derived highest level of education variable [32], and equivalized household income was categorized into quartiles, with the lowest quartile as the reference category and with a separate category for missing data on household income. Working and retirement status were categorized as employed (reference) versus non-employed and retired (reference) versus not retired, respectively. Marital status was dichotomized as living with a partner (reference) or not. Lifestyle factors included alcohol consumption grouped into once a month (reference), weekly, daily, or abstention [33], and smoking status was classified as non-smoker (reference), former smoker, and current smoker. Body mass index (BMI) was grouped into normal weight (including underweight as this was reported by only few respondents), overweight (BMI ≥ 25 kg/m^2^), and obesity (≥30 kg/m^2^) using WHO cut-off points [34]. Self-reported diabetes, hypertension, and cancer were classified as present or absent (reference). Depressive symptoms were assessed using the CES-D 10 (cut-off ≥10), with a score <10 as the reference [35]. Nutritional risk was evaluated with the AB-SCREEN II, where scores <38 indicated high risk, and low risk served as the reference. [36]. Social support was measured with the Medical Outcome Study Survey, with scores less than 4 were categorized as low (reference), and ≥4 as high [37]. Hearing was assessed through the question, "How would you rate your hearing, including with a hearing aid if you use one?" Responses were dichotomized into good (including excellent, very good, good; reference category) or poor (fair, poor). We also adjusted models by follow-up time, and baseline z-scores of each cognitive construct.

### 2.5 Statistical analysis

To detect latent groups of respondents with comparable movement activity patterns, we performed a LCA using as indicators the categorical variables of time spent sitting, walking, MPA, VPA, and sleep. These variables were treated as nominal in the analysis, allowing the LCA model to freely estimate response probabilities without imposing an ordinal structure. Information criteria, entropy, and interpretability of the latent classes were considered to determine the best model [38]. Specifically, lower Akaike Information Criterion (AIC), and Bayesian Information Criterion (BIC), and higher entropy indicate better model fit [38]. We assessed models ranging from 1 to 10 classes for both goodness of fit and substantive relevance. The LCA was performed using 10 different sets of random starting values to enhance robustness of the results, and each model underwent 100 iterations. After determining the optimal number of classes, participants were subsequently assigned to the latent classes based on maximum posterior probabilities.

To account for the small numbers of missing data in covariates (Appendix C), we used Multiple Imputation by Chained Equations, using all available variables at baseline and follow-up as predictors to generate 10 imputed datasets.

Differences in categorical covariates among movement activity groups were assessed using Chi-square tests, while continuous covariates were analyzed with t-tests for two groups or ANOVA for multiple groups. To account for multiple comparisons, the Bonferroni correction was applied.

Finally, we conducted multivariable linear regression analyses to examine the associations between the latent classes of movement activity patterns and the reliable change z-scores of memory, executive functioning and overall cognition.

The significance level was set at *p* < 0.05 and all statistical analyses were performed in Stata 18 (StataCorp. 2020, College Station, TX.), while figures were made with R 4.4.1

## 3 Results

### 3.1 Characteristics of the study population

The study population had an average follow-up period of 3 years (SD = 0.29, range: 1.3–5.6 years), a mean age of 62.1 years (SD = 10.0), and included 54.2% female. For more details see “Total sample” column of Table 1.

**Table 1:** Descriptive characteristics of the total sample at baseline and differences between the identified classes of movement activity patterns.

Characteristics^a^	Total sample	Sedentary/ disturbed sleep	Intermediately active/ normal sleep	Active/ normal sleep	p- value
	*n* = 12,212	*n* = 6584	*n* = 4229	*n* = 1399	
Age (years)	62.1 ± 10.0	63.2 ± 10.1	61.1 ± 9.8	59.6 ± 9.0	<0.01
Sex					<0.01
Female	54.2	54.8	55.5	47.5	
Education					<0.01
High	48.5	42.6	54.5	58.3	
Middle	47.2	51.7	43.0	39.2	
Low	4.3	5.7	2.6	2.5	
Equivalized household income (CAD)					<0.01
<35,000	15.7	18.4	13.7	9.4	
≥35,000.0–<53,033	15.5	17.0	14.3	12.3	
≥53,033–<88,388	39.0	37.3	40.2	43.3	
≥88,388.3	24.5	21.4	27.2	30.9	
Not provided data	5.3	5.9	4.7	4.1	
Marital status					<0.01
Not living with a partner	28.9	31.2	26.8	25.0	
Alcohol frequency					<0.01
Once a month	29.5	33.3	25.5	23.7	
Once a week, less than everyday	44.5	40.2	48.5	52.1	
Every day	15.8	15.1	17.0	16.1	
Abstainers	10.2	11.4	9.1	8.2	
Smoking status					<0.01
Never smoker	50.5	47.7	54.0	53.0	
Former smoking	42.2	42.9	41.5	41.2	
Currently smoking	7.3	9.5	4.5	5.9	
Working status					<0.01
Employed	54.1	50.1	58.0	60.8	
Retirement status					<0.01
Completely or partly retired	52.7	56.2	49.0	47.2	
Body mass index					<0.01
Normal	31.7	25.2	38.0	43.3	
Overweight	40.0	40.1	40.1	40.0	
Obesity	28.3	34.8	21.9	16.7	
Diabetes	16.1	19.1	13.4	10.0	<0.01
Hypertension	34.6	39.6	30.0	24.9	<0.01
Current depression	13.3	16.4	10.2	7.6	<0.01
History of cancer	10.8	11.7	10.2	9.0	<0.01
At high nutritional risk	31.9	38.1	25.6	21.6	<0.01
Low social support (score <4)	25.0	28.2	21.6	20.2	<0.01
Fair or poor hearing rate	9.7	11.1	8.4	7.4	<0.01
Cognitive constructs at baseline					
Memory	−0.01 ± 1.0	−0.06 ± 1.0	0.05 ± 1.0	0.00 ± 1.0	<0.01
Executive functioning	−0.01 ± 1.0	−0.07 ± 1.0	0.06 ± 1.0	0.04 ± 1.0	<0.01
Overall cognition	−0.01 ± 1.0	−0.07 ± 1.0	0.07 ± 1.0	0.03 ± 1.0	<0.01

a Categorical variables are reported as frequencies and continuous variables are reported as means with standard deviation.

### 3.2 Patterns of movement activities

We analyzed models with 2, 3 and 4 classes, as models incorporating more than 4 classes did not achieve convergence. Based on model fit parameters (Table 2), the main choice was between the three- and four-class solutions. The three-class model had a similar AIC to the four-class model and the lowest BIC. The four-class model had the highest entropy. We chose for the three-class solution as results were easier to interpret. The characteristics of each of the three classes are described below (Table 3):

• Class 1 (53.9%): “Sedentary/disturbed sleep”- This group showed the highest frequency of leisure sitting for ≥3 h/day (50.8%) and reported more often 0 h/day in walking, MPA, and VPA than the other classes. Participants in this class also had the highest frequencies of both short (43.6%) and long (8.0%) sleep.
• Class 2 (34.6%): “Intermediately active/normal sleep”- Characterized by a higher proportion of leisure sitting for 2−3 hours/day (50.4%). This group reported >0 to <1 h/day of walking and spent >0 to <1 h/day in MPA and VPA. Additionally, 68.6% of participants in this group reported normal sleep.
• Class 3 (11.5%): “Active/normal sleep”- This group had the highest prevalence of spending 0 to <2 h/day in leisure sitting activities (52.6%) and ≥1 h/day in walking, MPA, and VPA (53%, 24.7%, and 64.8% respectively). It also had the highest proportion of participants reporting normal sleep (74.5%) among the three groups.

**Table 2:** Model fit indicators for latent class solutions with different numbers of classes.

Model	Number of observations	Log likelihood	df	AIC	BIC	Entropy
2 classes	12,212	−54,869.39	21	109,781	109,936	0.847
3 classes	12,212	−54,813.79	32	109,692	109,929	0.861
4 classes	12,212	−54,799.18	43	109,684	110,003	0.916

df- degrees of freedom. AIC- Akaike information criterion. BIC- Bayesian information criterion.

**Table 3:** Movement characteristics of latent classes.

Data is expressed in frequencies. The predominant activity dimension for each class is highlighted in a more vivid red.

Table 1 presents the distribution of background characteristics for the three groups. Participants belonging to the “Sedentary/disturbed sleep” group were on average older and more often female, compared to those in the other groups. Additionally, participants in this group more frequently had risk factors for cognitive decline (Table 1). Participants in the “Active/normal sleep” group had a slightly better risk profile for cognitive decline compared to those in the “Intermediately active/normal sleep” group, but these differences were small and considered not clinically relevant (see Appendix D).

Post-hoc tests indicated that participants in the “Sedentary/disturbed sleep” group had the worst cognitive profile, with all domains significantly worse compared to those in the “Intermediately active/normal sleep” group and regarding executive functioning and overall cognitive compared to those in the “Active/normal sleep” group (see Appendix D).

### 3.3 Associations between patterns of movement activities and cognitive outcomes

**Fig. 2:**
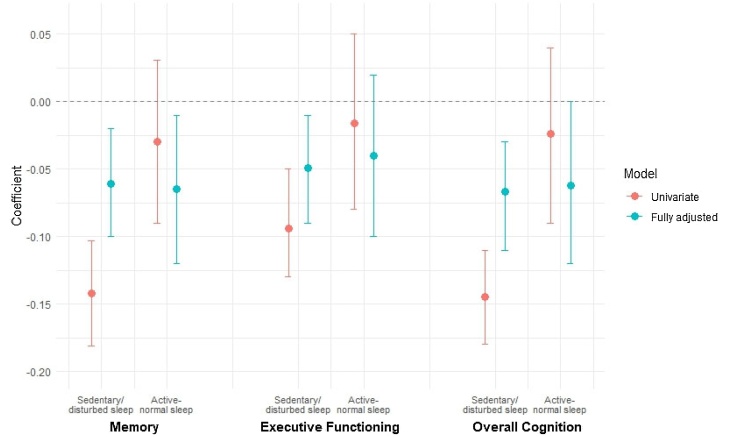
Univariate and fully adjusted regression estimates of movement activity patterns on cognitive change scores. Estimates are shown for Class 1 (Sedentary/disturbed sleep) and Class 3 (Active/normal sleep), using Class 2 (Intermediately active/normal sleep) as the reference group. Fully adjusted models control for variables listed in Table 1 and for duration to follow-up. See Appendix E for detailed results.

Considering that the best cognitive profile was found in class 2 “Intermediately active/normal sleep”, this one was considered as reference for the multivariable regression analysis (Fig. 2). Compared to this reference group, the other two groups showed significantly greater cognitive decline across all domains, except for executive functioning, where the “Intermediately active/normal sleep” group did not differ from the “Active/normal sleep” class (β = −0.040, 95%CI −0.103, 0.023). No significant differences in cognitive decline were observed between the “Sedentary/disturbed sleep” and the “Active/normal sleep” group across any cognitive domain.

For full results of the uni- and multivariable regression models, we refer to Appendix E.

One potential explanation why the “Active/normal sleep” group has a similar cognitive decline as “Sedentary/disturbed sleep” group, is that the former are more likely to be employed and might spend much of their working time sitting. However, sedentary work is not included in our measure of sedentary activities. To test whether the differences between classes differed between those employed and those not employed, we performed a sensitivity analysis, in which we added interactions between employment status and class membership to see whether the associations between movement activity patterns and cognitive decline differed between those with and without employment. However, these interactions were non-significant, suggesting that the patterns do not differ by employment status (see Appendix F).

## 4 Discussion

This study aimed to identify distinct groups of participants based on similar patterns of time spent in sedentary behavior, physical activity (walking, moderate, and vigorous), and sleep; examine their sociodemographic and lifestyle characteristics; and estimate the associations between these groups and changes in memory, executive functioning, and overall cognition. We identified three distinct groups. The largest group, “Sedentary/disturbed sleep”, comprised slightly over half of our sample and was characterized by a sedentary lifestyle and disturbed sleep. About one-third of respondents were in the “Intermediately active/normal sleep” class, characterized by normal sleep and intermediately levels of walking and light/moderate and vigorous activity. The smallest group, “Active/normal sleep”, accounted for approximately one-sixth of respondents and showed the highest levels of walking, light/moderate and vigorous activity with normal sleep. The “Sedentary/disturbed Sleep” group was older, predominantly female, and had a higher prevalence of cognitive decline risk factors, including chronic diseases, smoking, obesity, low social support, and poor hearing, compared to the other groups. Multivariable linear regression analyses showed that both the “Sedentary/disturbed sleep” and the “Active/normal sleep” classes showed more cognitive decline over a three-year period than the “Intermediately active/normal sleep” class.

To our knowledge, this study is the first to apply LCA to identify daily activity patterns and their associations with cognitive outcomes in a longitudinal design. A key finding is that the “Sedentary/disturbed sleep” group showed significantly greater overall cognitive decline than the other groups. This is in line with previous studies despite variations in how movement activities were assessed, such as the research from Zhang et al. and Miao et al. who classified participants based on self-reported engagement (yes/no) in various activities without considering intensity or duration, or the one from Li et al. that used the Global Physical Activity Questionnaire (GPAQ) to evaluate physical activity and sedentary behavior in a nationally representative U.S. sample. Despite these differences, all studies consistently found that more active groups showed better cognitive outcomes compared to inactive or sedentary groups. For instance, Zhang et al. identified four physically active classes that demonstrated better cognitive performance than the “Non-active” class [16]. Likewise, Miao et al. reported that among three lifestyle patterns, only the “active ageing” class was associated with a lower risk of mild cognitive impairment, while the “leisure” and “work-centered” patterns were not [15]. However, these studies were cross-sectional and did not account for sleep duration.

Contrary to naive assumptions that increased physical activity could result in greater cognitive benefits, the “Active/normal sleep” group showed greater cognitive decline than the “Intermediately active/normal sleep group”. While some studies using traditional statistical approaches report a dose-response relationship between physical activity and cognitive health [39,40], our study applied a more holistic, pattern-based approach using LCA, offering a different perspective. Similar to our findings, other LCA-based studies have shown that higher activity levels were sometimes associated with poorer cognition or no significant effects [41,42]. For example, Ringin et al. found that individuals engaging in high levels of physical activity had poorer cognitive scores than those with low or moderate levels of activity [41]. Similarly, Wu et al. reported that vigorous physical activity was not significantly associated with global cognition, episodic memory, or mental intactness [42]. One potential explanation is that the “Intermediately active/normal sleep” group is doing better not because they are less physically active than the “Active/normal sleep” group, but because they are performing cognitively stimulating activities at work. To examine this issue, we performed a sensitivity analysis adding interaction terms between latent classes and employment status. However, these results did not differ by employment status. Nonetheless, future research would profit from including information on sedentary behavior at work. Several explanations have been proposed for why physical activity may become detrimental for cognitive ageing above a certain level, i.e. the neural noise caused by exercise-induced catecholamines, stress-related increases in cortisol levels, and the activation of the limbic system linked to emotions like anxiety, all of which may negatively affect cognitive performance [43]. Currently, guidelines advice only a minimal amount of physical activity to remain cognitive healthy [44]. Further research on the potential negative effects of physical activity when surpassing a specific limit is needed to provide adequate guidance.

Short and long sleep, which were predominantly in the “Sedentary behavior/disturbed sleep” group, have both been associated with poorer brain health and cognitive decline in cohort studies [[45], [46], [47]]. Proposed mechanisms that may link abnormalities in sleep duration with accelerated cognitive decline include insufficient toxic metabolite clearance, neuroinflammation, increased oxidative stress, structural brain changes, and neurotransmitter impairments [45]. Given the clustering of sedentary behavior and abnormal sleep duration, it seems preferable to combine interventions to enhance physical activity and to optimize sleep duration. This is fully in line with multimodal interventions to improve cognitive ageing as for example studied within the FINGER initiative [48].

This study has several strengths, including a large sample size with a three-year follow-up and the joint consideration of sleep duration and movement activities, which provides deeper insights into daily activity patterns. Additionally, the neuropsychological battery used in the CLSA to assess cognition was carefully selected to capture cognitive changes in this cohort study. While these tests are not as commonly used as the Mini-Mental State Examination or the Montreal Cognitive Assessment, they have demonstrated comparability to other studies, particularly when assessing cognitive domains with the REY I, REY II, and VFT [23]. Nonetheless, some limitations should be addressed. Although we used well-validated questionnaires to assess sleep and physical activity, reliance on self-reported data may lead to inaccuracies in activity measurement [49]. Moreover, we assessed overall leisure sedentary time without distinguishing between cognitively passive and active sedentary activities, which have different associations with cognitive function and may contribute to inconsistencies in the literature regarding total sedentary time and cognitive decline [50]. Future research should explore these different dimensions of sedentary behavior and consider them in daily activity patterns. Some of these limitations could be mitigated by using accelerometers, which provide a more accurate measure of daily activities without relying on participant memory. However, these devices also have drawbacks, including higher study costs, variability in brand-specific algorithms, lack of standardized data analysis procedures, and participant burden, as they often require self-reported data to identify specific activities [51,52]. Given that dementia can have a prodromal phase of 10–15 years, we cannot rule out the possibility that some participants, particularly those in the Sedentary/disturbed sleep class, may have already exhibited early, undetected dementia pathology. Finally, generalizability may be negatively affected by the observed selection bias towards a healthier population. Compared to those who dropped out, participants included in the analyses were slightly less sedentary and more likely to engage in light/moderate and vigorous physical activity, while no significant differences were observed in sleep duration between the groups. This may have led to an underrepresentation of individuals with lower activity levels, potentially limiting the identification of the most sedentary profiles in the latent class structure. Since cognitive decline is probably stronger among those who were excluded or dropped out, our findings probably present conservative estimates.

Our findings show that persons with a pattern of moderate sitting time, moderate physical activity, and normal sleep duration show less cognitive decline than those with either a sedentary lifestyle and abnormal sleep duration or those with a very active movement pattern and normal sleep, suggesting that a balanced pattern of moderate activity and normal sleep may protect against accelerated cognitive decline.

## Funding

The first author acknowledges the fellowship received from the Consejo Nacional de Humanidades, Ciencias y Tecnologías (CONAHCYT) scholarship (2021-000007-01EXTF-00176), and the fellowship received from University Medical Center Groningen, University of Groningen.

## Disclaimers

The opinions expressed in this article are the author's own and do not reflect the views of the Canadian Longitudinal Study on Aging.

## Data availability statement

Data are available from the Canadian Longitudinal Study on Aging (www.clsa-elcv.ca) for researchers who meet the criteria for access to de-identified CLSA data.

## CRediT authorship contribution statement

**R.A. Palazuelos-González:** Writing - original draft, Methodology, Funding acquisition, Formal analysis, Conceptualization. **R.C. Oude-Voshaar:** Writing - review & editing, Supervision, Project administration, Methodology, Funding acquisition, Conceptualization. **N. Smidt:** Writing - review & editing, Supervision, Project administration, Methodology, Funding acquisition, Conceptualization. **A.C. Liefbroer:** Writing - review & editing, Supervision, Project administration, Methodology, Conceptualization.

## Declaration of competing interest

The authors declared no potential conflicts of interest with respect to the research, authorship, and/or publication of this article.

## Footnotes

Appendix A Supplementary material related to this article can be found, in the online version, at doi:https://doi.org/10.1016/j.jnha.2025.100619.
