# Supplementary material for: Patterns of time spent in sedentary behavior, physical activity, and sleep are associated with cognitive decline among CLSA participants: A latent class analysis
Source: J Nutr Health Aging. 2025 Jun 27;29(8):100619. doi: 10.1016/j.jnha.2025.100619 (PMC12268057; doi:10.1016/j.jnha.2025.100619)
Supplement: Supplementary file 1 [file mmc1.docx]

**Supplementary Materials**

Appendix A. Comparison of respondents with and without complete baseline data (see Figure 1 for reasons of non-selection)

|  | With complete baseline data | Without complete baseline data | p- value |
| --- | --- | --- | --- |
|  | n=17,184 | n=12,913 |  |
| Mean age (SD) | 62.8 (10.2) | 62.8 (10.3) | 0.742 |
| Sex |  |  |  |
| Women | 55.0 | 45.4 | <0.001 |
| Men | 45.0 | 54.6 |  |
| Education |  |  |  |
| High | 46.3 | 43.6 | <0.001 |
| Middle | 48.6 | 50.3 |  |
| Low | 5.1 | 6.1 |  |
| Equivalized household income |  |  |  |
| <34999.99 | 17.5 | 18.8 | <0.001 |
| 35000 - 53033.0001 | 16.3 | 14.8 |  |
| 53033.001 - 88388.33 | 37.9 | 33.0 |  |
| >=88388.34 | 22.6 | 18.5 |  |
| Not provided data | 5.8 | 14.9 |  |
| Marital status |  |  |  |
| Not living with a partner | 30.5 | 32.9 | <0.001 |
| Living with a partner | 69.5 | 67.1 |  |
| Alcohol frequency |  |  |  |
| Once a month | 30.0 | 29.4 | 0.004 |
| Once a week, less than everyday | 42.8 | 41.7 |  |
| Every day | 16.0 | 16.5 |  |
| Abstainers | 11.2 | 12.4 |  |
| Smoking status |  |  |  |
| Never smoker | 49.7 | 44.2 | <0.001 |
| Former smoking | 42.5 | 45.3 |  |
| Currently smoking | 7.8 | 10.6 |  |
| Working status |  |  |  |
| Employed | 51.4 | 50.6 | 0.204 |
| Unemployed | 48.6 | 49.4 |  |
| Retirement status |  |  |  |
| Completely and partly retired | 55.0 | 54.9 | 0.850 |
| Not retired | 45.0 | 45.1 |  |
| Body mass index |  |  |  |
| Normal | 31.2 | 28.9 | <0.001 |
| Overweight | 40.2 | 40.6 |  |
| Obesity | 28.6 | 30.6 |  |
| Diabetes | 16.9 | 18.7 | <0.001 |
| Hypertension | 35.7 | 38.0 | <0.001 |
| Depression | 14.3 | 19.0 | <0.001 |
| Cancer | 12.0 | 13.0 | 0.010 |
| Nutritional risk |  |  |  |
| Not at high nutritional risk | 66.4 | 60.9 |  |
| At high nutritional risk | 33.6 | 39.1 | <0.001 |
| Social support |  |  |  |
| Low support (score <4) | 26.0 | 30.5 | <0.001 |
| High support (score ≥4) | 74.0 | 69.5 |  |
| Hearing rate |  |  |  |
| Excellent, very good, good | 89.8 | 87.0 | <0.001 |
| Fair and poor | 10.2 | 13.0 |  |

Data is presented in frequencies unless indicated otherwise

Appendix B. Comparison between the final analytic sample and drop-out after baseline (see Figure 1 for reasons of drop-out)

|  | Final sample | Drop-out | p- value |
| --- | --- | --- | --- |
|  | n= 12,212 | n=4,792 |  |
| Mean age (SD) | 62.1 (10.0) | 64.4 (10.5) | <0.001 |
| Sex |  |  |  |
| Women | 54.2 | 56.9 | 0.001 |
| Men | 45.8 | 43.1 |  |
| Education |  |  |  |
| High | 48.5 | 40.9 | <0.001 |
| Middle | 47.2 | 52.1 |  |
| Low | 4.3 | 7.0 |  |
| Equivalized household income |  |  |  |
| <34999.99 | 15.7 | 21.8 | <0.001 |
| 35000 - 53033.0001 | 15.5 | 18.1 |  |
| 53033.001 - 88388.33 | 39.0 | 35.2 |  |
| >=88388.34 | 24.5 | 17.9 |  |
| Not provided data | 5.3 | 7.1 |  |
| Marital status |  |  |  |
| Not living with a partner | 28.9 | 34.3 | <0.001 |
| Living with a partner | 71.1 | 65.7 |  |
| Alcohol frequency |  |  |  |
| Once a month | 29.5 | 31.3 | <0.001 |
| Once a week, less than everyday | 44.5 | 38.6 |  |
| Every day | 15.8 | 16.6 |  |
| Abstainers | 10.2 | 13.5 |  |
| Smoking status |  |  |  |
| Never smoker | 50.5 | 47.7 | <0.001 |
| Former smoking | 42.2 | 43.2 |  |
| Currently smoking | 7.3 | 9.1 |  |
| Working status |  |  |  |
| Employed | 54.0 | 44.9 | <0.001 |
| Unemployed | 46.0 | 55.1 |  |
| Retirement status |  |  |  |
| Completely and partly retired | 52.6 | 60.8 | <0.001 |
| Not retired | 47.4 | 39.2 |  |
| Body mass index |  |  |  |
| Normal | 31.7 | 30.1 | 0.085 |
| Overweight | 40.1 | 40.4 |  |
| Obesity | 28.3 | 29.5 |  |
| Diabetes | 16.1 | 18.8 | <0.001 |
| Hypertension | 34.6 | 38.3 | <0.001 |
| Depression | 13.3 | 17.0 | <0.001 |
| Cancer | 10.8 | 14.8 | <0.001 |
| Nutritional risk |  |  |  |
| Not at high nutritional risk | 68.2 | 62.1 | <0.001 |
| At high nutritional risk | 31.8 | 37.9 |  |
| Social support |  |  |  |
| Low support (score <4) | 24.9 | 28.7 | <0.001 |
| High support (score ≥4) | 75.1 | 71.3 |  |
| Hearing rate |  |  |  |
| Excellent, very good, good | 90.3 | 88.5 | <0.001 |
| Fair and poor | 9.7 | 11.5 |  |
| Sitting |  |  |  |
| 0 to <2 hour/day | 22.4 | 20.0 | <0.001 |
| 2 to <3 hour/day | 41.2 | 40.9 |  |
| ≥ 3 hour/day | 36.5 | 39.1 |  |
| Walking |  |  |  |
| 0 hour/day | 14.0 | 16.3 | <0.001 |
| 0- <1 hour/day | 63.7 | 61.0 |  |
| ≥ 1 hour/day | 22.3 | 22.7 |  |
| Light/moderate |  |  |  |
| 0 hour/day | 67.6 | 71.1 | <0.001 |
| 0- <1 hour/day | 26.1 | 23.1 |  |
| ≥ 1 hour/day | 6.3 | 5.8 |  |
| Vigorous |  |  |  |
| 0 hour/day | 51.8 | 57.0 | <0.001 |
| 0- <1 hour/day | 39.1 | 35.5 |  |
| ≥ 1 hour/day | 9.1 | 7.5 |  |
| Sleep |  |  |  |
| 7- hour/day | 58.4 | 57.8 | 0.375 |
| <7 hour/day | 36.5 | 36.6 |  |
| >8 hour/day | 5.1 | 5.7 |  |
|  |  |  |  |

Data is presented in frequencies unless indicated otherwise

Appendix C. Overview of Missing Data Across Variables

|  | Missing (n) |
| --- | --- |
| Age | 0 |
| Sex | 0 |
| Education | 0 |
| Equivalized household income | 0 |
| Marital status | 2 |
| Alcohol frequency | 259 |
| Smoking status | 0 |
| Working status | 36 |
| Retirement status | 34 |
| Body mass index | 21 |
| Diabetes | 0 |
| Hypertension | 0 |
| Depression | 0 |
| Cancer | 0 |
| Nutritional risk | 97 |
| Social support | 144 |
| Hearing rate | 7 |

Appendix D. Post-hoc comparisons between classes of movement activities

Bonferroni correction- α adjusted=0.05/3= ≈0.017

|  | Between Class 1 and 2 | |  |  | Between Class 1 and 3 | |  |  | Between Class 2 and 3 | |  |
| --- | --- | --- | --- | --- | --- | --- | --- | --- | --- | --- | --- |
|  | Class 1 | Class 2 | p- value |  | Class 1 | Class 3 | p- value |  | Class 2 | Class 3 | p- value |
|  | n=6,584 | n=4,229 |  |  | n=6,584 | n=1,399 |  |  | n=4,229 | n=1,399 |  |
| Age (mean ± SD) | 63.2 ± 10.1 | 61.1 ± 9.8 | <0.01 |  | 63.2 ± 10.1 | 59.6 ± 9.0 | <0.01 |  | 61.1 ± 9.8 | 59.6 ± 9.0 | <0.01 |
| Sex |  |  |  |  |  |  |  |  |  |  |  |
| Women | 54.8 | 55.5 | 0.507 |  | 54.8 | 47.5 | <0.01 |  | 55.5 | 47.5 | <0.01 |
| Men | 45.2 | 44.6 |  |  | 45.2 | 52.5 |  |  | 44.6 | 52.5 |  |
| Education |  |  |  |  |  |  |  |  |  |  |  |
| High | 42.6 | 54.5 | <0.01 |  | 42.6 | 58.3 | <0.01 |  | 54.5 | 58.3 | 0.039 |
| Middle | 51.7 | 43.0 |  |  | 51.7 | 39.2 |  |  | 43.0 | 39.2 |  |
| Low | 5.7 | 2.6 |  |  | 5.7 | 2.5 |  |  | 2.6 | 2.5 |  |
| Equivalized household income |  |  |  |  |  |  |  |  |  |  |  |
| <34999.99 | 18.4 | 13.7 | <0.01 |  | 18.4 | 9.4 | <0.01 |  | 13.7 | 9.4 | <0.01 |
| 35000 - 53033.0001 | 17.0 | 14.3 |  |  | 17.0 | 12.3 |  |  | 14.3 | 12.3 |  |
| 53033.001 - 88388.33 | 37.3 | 40.2 |  |  | 37.3 | 43.3 |  |  | 40.2 | 43.3 |  |
| >=88388.34 | 21.4 | 27.2 |  |  | 21.4 | 30.9 |  |  | 27.2 | 30.9 |  |
| Not provided data | 5.9 | 4.7 |  |  | 5.9 | 4.1 |  |  | 4.7 | 4.1 |  |
| Marital status |  |  |  |  |  |  |  |  |  |  |  |
| Not living with a partner | 31.2 | 26.8 | <0.01 |  | 31.2 | 25.0 | <0.01 |  | 26.8 | 25.0 | 0.202 |
| Living with a partner | 68.8 | 73.3 |  |  | 68.8 | 75.0 |  |  | 73.3 | 75.0 |  |
| Alcohol frequency |  |  |  |  |  |  |  |  |  |  |  |
| Once a month | 33.3 | 25.5 | <0.01 |  | 33.3 | 23.7 | <0.01 |  | 25.5 | 23.7 | 0.122 |
| Once a week, less than everyday | 40.2 | 48.5 |  |  | 40.2 | 52.1 |  |  | 48.5 | 52.1 |  |
| Every day | 15.1 | 17.0 |  |  | 15.1 | 16.1 |  |  | 17.0 | 16.1 |  |
| Abstainers | 11.4 | 9.1 |  |  | 11.4 | 8.2 |  |  | 9.1 | 8.2 |  |
| Smoking status |  |  |  |  |  |  |  |  |  |  |  |
| Never smoker | 47.7 | 54.0 | <0.01 |  | 47.7 | 53.0 | <0.01 |  | 54.0 | 53.0 | 0.106 |
| Former smoking | 42.9 | 41.5 |  |  | 42.9 | 41.2 |  |  | 41.5 | 41.2 |  |
| Currently smoking | 9.5 | 4.5 |  |  | 9.5 | 5.9 |  |  | 4.5 | 5.9 |  |
| Working status |  |  |  |  |  |  |  |  |  |  |  |
| Employed | 50.1 | 58.0 | <0.01 |  | 50.1 | 60.8 | <0.01 |  | 58.0 | 60.8 | 0.064 |
| Unemployed | 49.9 | 42.0 |  |  | 49.9 | 39.2 |  |  | 42.0 | 39.2 |  |
| Retirement status |  |  |  |  |  |  |  |  |  |  |  |
| Completely and partly retired | 56.2 | 49.0 | <0.01 |  | 56.2 | 47.2 | <0.01 |  | 49.0 | 47.2 | 0.278 |
| Not retired | 43.8 | 51.0 |  |  | 43.8 | 52.8 |  |  | 51.0 | 52.8 |  |
| Body mass index |  |  |  |  |  |  |  |  |  |  |  |
| Normal | 25.2 | 38.0 | <0.01 |  | 25.2 | 43.3 | <0.01 |  | 38.0 | 43.3 | <0.01 |
| Overweight | 40.1 | 40.1 |  |  | 40.1 | 40.0 |  |  | 40.1 | 40.0 |  |
| Obesity | 34.8 | 21.9 |  |  | 34.8 | 16.7 |  |  | 21.9 | 16.7 |  |
| Diabetes |  |  |  |  |  |  |  |  |  |  |  |
| No | 80.9 | 86.6 | <0.01 |  | 80.9 | 90.0 | <0.01 |  | 86.6 | 90.0 | <0.01 |
| Yes | 19.1 | 13.4 |  |  | 19.1 | 10.0 |  |  | 13.4 | 10.0 |  |
| Hypertension |  |  |  |  |  |  |  |  |  |  |  |
| No | 60.4 | 70.0 | <0.01 |  | 60.4 | 75.1 | <0.01 |  | 70.0 | 75.1 | <0.01 |
| Yes | 39.6 | 30.0 |  |  | 39.6 | 24.9 |  |  | 30.0 | 24.9 |  |
| Depression |  |  |  |  |  |  |  |  |  |  |  |
| No | 83.6 | 89.8 | <0.01 |  | 83.6 | 92.4 | <0.01 |  | 89.8 | 92.4 | <0.01 |
| Yes | 16.4 | 10.2 |  |  | 16.4 | 7.6 |  |  | 10.2 | 7.6 |  |
| Cancer |  |  |  |  |  |  |  |  |  |  |  |
| No | 88.3 | 89.8 | 0.017 |  | 88.3 | 91.0 | <0.01 |  | 89.8 | 91.0 | 0.198 |
| Yes | 11.7 | 10.2 |  |  | 11.7 | 9.0 |  |  | 10.2 | 9.0 |  |
| Nutritional risk |  |  |  |  |  |  |  |  |  |  |  |
| Not at high nutritional risk | 61.9 | 74.4 | <0.01 |  | 61.9 | 78.4 | <0.01 |  | 74.4 | 78.4 | <0.01 |
| At high nutritional risk | 38.1 | 25.6 |  |  | 38.1 | 21.6 |  |  | 25.6 | 21.6 |  |
| Social support |  |  |  |  |  |  |  |  |  |  |  |
| Low support score <4 ) | 28.2 | 21.6 | <0.01 |  | 28.2 | 20.2 | <0.01 |  | 21.6 | 20.2 | 0.318 |
| High support (score ≥4) | 71.8 | 78.4 |  |  | 71.8 | 79.8 |  |  | 78.4 | 79.8 |  |
| Hearing rate |  |  |  |  |  |  |  |  |  |  |  |
| Excellent, very good, good | 88.9 | 91.6 | <0.01 |  | 88.9 | 92.6 | <0.01 |  | 91.6 | 92.6 | 0.253 |
| Fair and poor | 11.1 | 8.4 |  |  | 11.1 | 7.4 |  |  | 8.4 | 7.4 |  |
| Memory z-score | -0.05 ± 1.0 | 0.05 ± 1.0 | <0.01 |  | -0.05 ± 1.0 | 0.00 ± 1.0 | 0.225 |  | 0.05 ± 1.0 | 0.00 ± 1.0 | 0.333 |
| Executive function z-scores | -0.07 ± 1.0 | 0.06 ± 1.0 | <0.01 |  | -0.07 ± 1.0 | 0.04 ± 1.0 | <0.01 |  | 0.06 ± 1.0 | 0.04 ± 1.0 | 1.000 |
| overall cognitive function z-scores | -0.07 ± 1.0 | 0.07 ± 1.0 | <0.01 |  | -0.07 ± 1.0 | 0.03 ± 1.0 | 0.002 |  | 0.07 ± 1.0 | 0.03 ± 1.0 | 0.467 |

Data is presented in frequencies unless indicated otherwise.

Appendix E. Linear uni- and multivariable regression results of movement activity patterns and a) memory, b) executive function, and c) overall cognition.

1. Memory

|  | Univariate | | | | Univariate | | | | Fully adjusted | | | | | Fully adjusted | | | | |
| --- | --- | --- | --- | --- | --- | --- | --- | --- | --- | --- | --- | --- | --- | --- | --- | --- | --- | --- |
|  |  | 95 CI |  |  |  | 95 CI |  |  |  | 95 CI |  |  |  | | 95 CI |  |  |  |
|  | Coef | Lower | Upper | p- value | Coef | Lower | Upper | p- value | Coef | Lower | Upper | p- value | Coef | | Lower | Upper | p- value |  |
| Sedentary/disturbed sleep | Ref |  |  |  | -0.142 | -0.181 | -0.103 | 0.000 | Ref |  |  |  | -0.061 | | -0.10 | -0.02 | 0.00 |  |
| Interm active-normal short sleep | 0.142 | 0.10 | 0.18 | 0.00 | Ref |  |  |  | 0.061 | 0.02 | 0.10 | 0.00 | Ref | |  |  |  |  |
| Active-normal sleep | 0.112 | 0.05 | 0.17 | 0.00 | -0.030 | -0.090 | 0.031 | 0.337 | -0.004 | -0.06 | 0.05 | 0.89 | -0.065 | | -0.12 | -0.01 | 0.03 |  |
| Memory at baseline | 0.006 | -0.01 | 0.02 | 0.49 |  |  |  |  | -0.011 | -0.03 | 0.01 | 0.23 |  | |  |  |  |  |
| Follow-up time | 0.015 | -0.05 | 0.08 | 0.63 |  |  |  |  | -0.073 | -0.13 | -0.01 | 0.02 |  | |  |  |  |  |
| Age | -0.018 | -0.02 | -0.02 | 0.00 |  |  |  |  | -0.016 | -0.02 | -0.01 | 0.00 |  | |  |  |  |  |
| Sex- Women | Ref |  |  |  |  |  |  |  | Ref |  |  |  |  | |  |  |  |  |
| Men | -0.100 | -0.14 | -0.06 | 0.00 |  |  |  |  | -0.118 | -0.16 | -0.08 | 0.00 |  | |  |  |  |  |
| Education- High | Ref |  |  |  |  |  |  |  | Ref |  |  |  |  | |  |  |  |  |
| Middle | -0.199 | -0.24 | -0.16 | 0.00 |  |  |  |  | -0.146 | -0.18 | -0.11 | 0.00 |  | |  |  |  |  |
| Low | -0.220 | -0.31 | -0.13 | 0.00 |  |  |  |  | -0.009 | -0.10 | 0.08 | 0.84 |  | |  |  |  |  |
| Eq household income- <34999.99 | Ref |  |  |  |  |  |  |  | Ref |  |  |  |  | |  |  |  |  |
| 35000 - 53033.0001 | 0.064 | 0.00 | 0.13 | 0.05 |  |  |  |  | 0.017 | -0.05 | 0.08 | 0.61 |  | |  |  |  |  |
| 53033.001 - 88388.33 | 0.190 | 0.14 | 0.24 | 0.00 |  |  |  |  | 0.077 | 0.02 | 0.13 | 0.01 |  | |  |  |  |  |
| >=88388.34 | 0.225 | 0.17 | 0.28 | 0.00 |  |  |  |  | 0.087 | 0.03 | 0.15 | 0.01 |  | |  |  |  |  |
| Not provided data | 0.003 | -0.09 | 0.09 | 0.95 |  |  |  |  | -0.015 | -0.10 | 0.07 | 0.74 |  | |  |  |  |  |
| Marital status- Not living with a partner | Ref |  |  |  |  |  |  |  | Ref |  |  |  |  | |  |  |  |  |
| Living with a partner | 0.112 | 0.07 | 0.15 | 0.00 |  |  |  |  | 0.021 | -0.02 | 0.07 | 0.35 |  | |  |  |  |  |
| Working status- Employed | Ref |  |  |  |  |  |  |  | Ref |  |  |  |  | |  |  |  |  |
| Unemployed | -0.225 | -0.26 | -0.19 | 0.00 |  |  |  |  | -0.055 | -0.11 | 0.00 | 0.04 |  | |  |  |  |  |
| Completely and partly retired | Ref |  |  |  |  |  |  |  | Ref |  |  |  |  | |  |  |  |  |
| Not retired | 0.223 | 0.19 | 0.26 | 0.00 |  |  |  |  | -0.059 | -0.11 | 0.00 | 0.04 |  | |  |  |  |  |
| Alcohol frequency- Once a month | Ref |  |  |  |  |  |  |  | Ref |  |  |  |  | |  |  |  |  |
| Once a week, less than everyday | 0.071 | 0.03 | 0.11 | 0.00 |  |  |  |  | 0.015 | -0.03 | 0.06 | 0.49 |  | |  |  |  |  |
| Every day | -0.010 | -0.07 | 0.05 | 0.72 |  |  |  |  | 0.020 | -0.04 | 0.08 | 0.50 |  | |  |  |  |  |
| Abstainers | -0.002 | -0.07 | 0.06 | 0.96 |  |  |  |  | 0.030 | -0.03 | 0.09 | 0.35 |  | |  |  |  |  |
| Smoking status- Never smoker | Ref |  |  |  |  |  |  |  | Ref |  |  |  |  | |  |  |  |  |
| Former smoking | -0.094 | -0.13 | -0.06 | 0.00 |  |  |  |  | -0.014 | -0.05 | 0.02 | 0.46 |  | |  |  |  |  |
| Currently smoking | -0.144 | -0.21 | -0.07 | 0.00 |  |  |  |  | -0.107 | -0.18 | -0.04 | 0.00 |  | |  |  |  |  |
| Body mass index- Normal | Ref |  |  |  |  |  |  |  | Ref |  |  |  |  | |  |  |  |  |
| Overweight | -0.121 | -0.16 | -0.08 | 0.00 |  |  |  |  | -0.055 | -0.10 | -0.01 | 0.01 |  | |  |  |  |  |
| Obesity | -0.134 | -0.18 | -0.09 | 0.00 |  |  |  |  | -0.054 | -0.10 | -0.01 | 0.03 |  | |  |  |  |  |
| Diabetes | -0.136 | -0.18 | -0.09 | 0.00 |  |  |  |  | -0.032 | -0.08 | 0.02 | 0.20 |  | |  |  |  |  |
| Hypertension | -0.160 | -0.20 | -0.12 | 0.00 |  |  |  |  | -0.030 | -0.07 | 0.01 | 0.14 |  | |  |  |  |  |
| Depression | -0.114 | -0.17 | -0.06 | 0.00 |  |  |  |  | -0.073 | -0.13 | -0.02 | 0.01 |  | |  |  |  |  |
| Cancer | -0.083 | -0.14 | -0.03 | 0.01 |  |  |  |  | 0.018 | -0.04 | 0.08 | 0.54 |  | |  |  |  |  |
| Nutritional risk- Not at high nutritional risk | Ref |  |  |  |  |  |  |  | Ref |  |  |  |  | |  |  |  |  |
| At high nutritional risk | -0.078 | -0.12 | -0.04 | 0.00 |  |  |  |  | -0.006 | -0.05 | 0.03 | 0.78 |  | |  |  |  |  |
| Social support- Low | Ref |  |  |  |  |  |  |  | Ref |  |  |  |  | |  |  |  |  |
| High support (score ≥4) | 0.110 | 0.07 | 0.15 | 0.00 |  |  |  |  | 0.035 | -0.01 | 0.08 | 0.12 |  | |  |  |  |  |
| Hearing rate- Excellent, very good, good | Ref |  |  |  |  |  |  |  | Ref |  |  |  |  | |  |  |  |  |
| Fair and poor | -0.156 | -0.22 | -0.10 | 0.00 |  |  |  |  | -0.056 | -0.12 | 0.00 | 0.06 |  | |  |  |  |  |

1. Executive function

|  | Univariate | | | | Univariate | | | | Fully adjusted | | | | | Fully adjusted | | | | |
| --- | --- | --- | --- | --- | --- | --- | --- | --- | --- | --- | --- | --- | --- | --- | --- | --- | --- | --- |
|  |  | 95 CI |  |  |  | 95 CI |  |  |  | 95 CI |  |  |  | | 95 CI |  |  |  |
|  | Coef | Lower | Upper | p- value | Coef | Lower | Upper | p- value | Coef | Lower | Upper | p- value | Coef | | Lower | Upper | p- value |  |
| Sedentary/disturbed sleep | Ref |  |  |  | -0.094 | -0.13 | -0.05 | 0.00 | Ref |  |  |  | -0.049 | | -0.09 | -0.01 | 0.02 |  |
| Interm active-normal short sleep | 0.094 | 0.05 | 0.13 | 0.00 | Ref |  |  |  | 0.049 | 0.01 | 0.09 | 0.02 | Ref | |  |  |  |  |
| Active-normal sleep | 0.078 | 0.02 | 0.14 | 0.01 | -0.016 | -0.08 | 0.05 | 0.63 | 0.009 | -0.05 | 0.07 | 0.77 | -0.040 | | -0.10 | 0.02 | 0.21 |  |
| Executive function at baseline | -0.029 | -0.05 | -0.01 | 0.00 |  |  |  |  | -0.058 | -0.08 | -0.04 | 0.00 |  | |  |  |  |  |
| Follow-up time | -0.013 | -0.08 | 0.05 | 0.70 |  |  |  |  | -0.055 | -0.12 | 0.01 | 0.10 |  | |  |  |  |  |
| Age | -0.007 | -0.01 | -0.01 | 0.00 |  |  |  |  | -0.007 | -0.01 | 0.00 | 0.00 |  | |  |  |  |  |
| Sex- Women | Ref |  |  |  |  |  |  |  | Ref |  |  |  |  | |  |  |  |  |
| Men | -0.025 | -0.06 | 0.01 | 0.19 |  |  |  |  | -0.051 | -0.09 | -0.01 | 0.01 |  | |  |  |  |  |
| Education- High | Ref |  |  |  |  |  |  |  | Ref |  |  |  |  | |  |  |  |  |
| Middle | -0.141 | -0.18 | -0.10 | 0.00 |  |  |  |  | -0.120 | -0.16 | -0.08 | 0.00 |  | |  |  |  |  |
| Low | -0.079 | -0.17 | 0.01 | 0.10 |  |  |  |  | 0.042 | -0.06 | 0.14 | 0.40 |  | |  |  |  |  |
| Eq household income- <34999.99 | Ref |  |  |  |  |  |  |  | Ref |  |  |  |  | |  |  |  |  |
| 35000 - 53033.0001 | 0.031 | -0.04 | 0.10 | 0.37 |  |  |  |  | 0.020 | -0.05 | 0.09 | 0.57 |  | |  |  |  |  |
| 53033.001 - 88388.33 | 0.091 | 0.04 | 0.15 | 0.00 |  |  |  |  | 0.037 | -0.02 | 0.09 | 0.22 |  | |  |  |  |  |
| >=88388.34 | 0.183 | 0.12 | 0.24 | 0.00 |  |  |  |  | 0.110 | 0.04 | 0.17 | 0.00 |  | |  |  |  |  |
| Not provided data | 0.006 | -0.09 | 0.10 | 0.90 |  |  |  |  | -0.008 | -0.10 | 0.09 | 0.87 |  | |  |  |  |  |
| Marital status- Not living with a partner | Ref |  |  |  |  |  |  |  | Ref |  |  |  |  | |  |  |  |  |
| Living with a partner | 0.078 | 0.04 | 0.12 | 0.00 |  |  |  |  | 0.005 | -0.04 | 0.05 | 0.84 |  | |  |  |  |  |
| Working status- Employed | Ref |  |  |  |  |  |  |  | Ref |  |  |  |  | |  |  |  |  |
| Unemployed | -0.101 | -0.14 | -0.06 | 0.00 |  |  |  |  | -0.050 | -0.10 | 0.00 | 0.07 |  | |  |  |  |  |
| Completely and partly retired | Ref |  |  |  |  |  |  |  | Ref |  |  |  |  | |  |  |  |  |
| Not retired | 0.073 | 0.04 | 0.11 | 0.00 |  |  |  |  | -0.080 | -0.14 | -0.02 | 0.01 |  | |  |  |  |  |
| Alcohol frequency- Once a month | Ref |  |  |  |  |  |  |  | Ref |  |  |  |  | |  |  |  |  |
| Once a week, less than everyday | 0.073 | 0.03 | 0.12 | 0.00 |  |  |  |  | 0.043 | 0.00 | 0.09 | 0.07 |  | |  |  |  |  |
| Every day | 0.080 | 0.02 | 0.14 | 0.01 |  |  |  |  | 0.090 | 0.03 | 0.15 | 0.00 |  | |  |  |  |  |
| Abstainers | -0.013 | -0.08 | 0.06 | 0.71 |  |  |  |  | 0.006 | -0.06 | 0.07 | 0.87 |  | |  |  |  |  |
| Smoking status- Never smoker | Ref |  |  |  |  |  |  |  | Ref |  |  |  |  | |  |  |  |  |
| Former smoking | -0.057 | -0.10 | -0.02 | 0.00 |  |  |  |  | -0.022 | -0.06 | 0.02 | 0.28 |  | |  |  |  |  |
| Currently smoking | -0.084 | -0.16 | -0.01 | 0.03 |  |  |  |  | -0.047 | -0.12 | 0.03 | 0.21 |  | |  |  |  |  |
| Body mass index- Normal | Ref |  |  |  |  |  |  |  | Ref |  |  |  |  | |  |  |  |  |
| Overweight | -0.072 | -0.12 | -0.03 | 0.00 |  |  |  |  | -0.037 | -0.08 | 0.01 | 0.11 |  | |  |  |  |  |
| Obesity | -0.055 | -0.10 | -0.01 | 0.02 |  |  |  |  | 0.012 | -0.04 | 0.06 | 0.64 |  | |  |  |  |  |
| Diabetes | -0.114 | -0.16 | -0.06 | 0.00 |  |  |  |  | -0.065 | -0.12 | -0.01 | 0.02 |  | |  |  |  |  |
| Hypertension | -0.085 | -0.12 | -0.05 | 0.00 |  |  |  |  | -0.029 | -0.07 | 0.01 | 0.18 |  | |  |  |  |  |
| Depression | -0.074 | -0.13 | -0.02 | 0.01 |  |  |  |  | -0.030 | -0.09 | 0.03 | 0.31 |  | |  |  |  |  |
| Cancer | -0.085 | -0.14 | -0.03 | 0.01 |  |  |  |  | -0.042 | -0.10 | 0.02 | 0.17 |  | |  |  |  |  |
| Nutritional risk- Not at high nutritional risk | Ref |  |  |  |  |  |  |  | Ref |  |  |  |  | |  |  |  |  |
| At high nutritional risk | -0.088 | -0.13 | -0.05 | 0.00 |  |  |  |  | -0.046 | -0.09 | 0.00 | 0.03 |  | |  |  |  |  |
| Social support- Low | Ref |  |  |  |  |  |  |  | Ref |  |  |  |  | |  |  |  |  |
| High support (score ≥4) | 0.091 | 0.05 | 0.13 | 0.00 |  |  |  |  | 0.045 | 0.00 | 0.09 | 0.06 |  | |  |  |  |  |
| Hearing rate- Excellent, very good, good | Ref |  |  |  |  |  |  |  | Ref |  |  |  |  | |  |  |  |  |
| Fair and poor | -0.057 | -0.12 | 0.01 | 0.08 |  |  |  |  | -0.004 | -0.07 | 0.06 | 0.89 |  | |  |  |  |  |

1. Overall cognition

|  | Univariate | | | | Univariate | | | | | Fully adjusted | | | | | Fully adjusted | | | | |
| --- | --- | --- | --- | --- | --- | --- | --- | --- | --- | --- | --- | --- | --- | --- | --- | --- | --- | --- | --- |
|  |  | 95 CI |  |  |  | 95 CI |  |  |  | | 95 CI |  |  |  | | 95 CI |  |  |  |
|  | Coef | Lower | Upper | p- value | Coef | Lower | Upper | p- value | Coef | | Lower | Upper | p- value | Coef | | Lower | Upper | p- value |  |
| Sedentary/disturbed sleep | Ref |  |  |  | -0.145 | -0.18 | -0.11 | 0.00 | Ref | |  |  |  | -0.067 | | -0.11 | -0.03 | 0.00 |  |
| Interm active-normal short sleep | 0.145 | 0.11 | 0.18 | 0.00 | Ref |  |  |  | 0.067 | | 0.03 | 0.11 | 0.00 | Ref | |  |  |  |  |
| Active-normal sleep | 0.121 | 0.06 | 0.18 | 0.00 | -0.024 | -0.09 | 0.04 | 0.44 | 0.004 | | -0.05 | 0.06 | 0.89 | -0.062 | | -0.12 | 0.00 | 0.04 |  |
| Overall cognition at baseline | -0.002 | -0.02 | 0.02 | 0.86 |  |  |  |  | -0.035 | | -0.05 | -0.02 | 0.00 |  | |  |  |  |  |
| Follow-up time | -0.001 | -0.06 | 0.06 | 0.98 |  |  |  |  | -0.088 | | -0.15 | -0.03 | 0.01 |  | |  |  |  |  |
| Age | -0.018 | -0.02 | -0.02 | 0.00 |  |  |  |  | -0.016 | | -0.02 | -0.01 | 0.00 |  | |  |  |  |  |
| Sex- Women | Ref |  |  |  |  |  |  |  |  | |  |  |  |  | |  |  |  |  |
| Men | -0.085 | -0.12 | -0.05 | 0.00 |  |  |  |  | -0.107 | | -0.15 | -0.07 | 0.00 |  | |  |  |  |  |
| Education- High | Ref |  |  |  |  |  |  |  |  | |  |  |  |  | |  |  |  |  |
| Middle | -0.190 | -0.23 | -0.15 | 0.00 |  |  |  |  | -0.141 | | -0.18 | -0.10 | 0.00 |  | |  |  |  |  |
| Low | -0.187 | -0.28 | -0.10 | 0.00 |  |  |  |  | 0.030 | | -0.06 | 0.12 | 0.53 |  | |  |  |  |  |
| Eq household income- <34999.99 | Ref |  |  |  |  |  |  |  |  | |  |  |  |  | |  |  |  |  |
| 35000 - 53033.0001 | 0.050 | -0.01 | 0.11 | 0.13 |  |  |  |  | 0.009 | | -0.06 | 0.07 | 0.78 |  | |  |  |  |  |
| 53033.001 - 88388.33 | 0.168 | 0.11 | 0.22 | 0.00 |  |  |  |  | 0.064 | | 0.01 | 0.12 | 0.03 |  | |  |  |  |  |
| >=88388.34 | 0.243 | 0.19 | 0.30 | 0.00 |  |  |  |  | 0.115 | | 0.05 | 0.18 | 0.00 |  | |  |  |  |  |
| Not provided data | -0.009 | -0.10 | 0.08 | 0.85 |  |  |  |  | -0.023 | | -0.11 | 0.07 | 0.62 |  | |  |  |  |  |
| Marital status- Not living with a partner | Ref |  |  |  |  |  |  |  |  | |  |  |  |  | |  |  |  |  |
| Living with a partner | 0.117 | 0.08 | 0.16 | 0.00 |  |  |  |  | 0.015 | | -0.03 | 0.06 | 0.50 |  | |  |  |  |  |
| Working status- Employed | Ref |  |  |  |  |  |  |  |  | |  |  |  |  | |  |  |  |  |
| Unemployed | -0.226 | -0.26 | -0.19 | 0.00 |  |  |  |  | -0.066 | | -0.12 | -0.01 | 0.01 |  | |  |  |  |  |
| Completely and partly retired | Ref |  |  |  |  |  |  |  |  | |  |  |  |  | |  |  |  |  |
| Not retired | 0.214 | 0.18 | 0.25 | 0.00 |  |  |  |  | -0.081 | | -0.14 | -0.02 | 0.01 |  | |  |  |  |  |
| Alcohol frequency- Once a month | Ref |  |  |  |  |  |  |  |  | |  |  |  |  | |  |  |  |  |
| Once a week, less than everyday | 0.083 | 0.04 | 0.13 | 0.00 |  |  |  |  | 0.030 | | -0.01 | 0.07 | 0.19 |  | |  |  |  |  |
| Every day | 0.019 | -0.04 | 0.08 | 0.50 |  |  |  |  | 0.055 | | 0.00 | 0.11 | 0.06 |  | |  |  |  |  |
| Abstainers | 0.002 | -0.06 | 0.07 | 0.95 |  |  |  |  | 0.033 | | -0.03 | 0.10 | 0.31 |  | |  |  |  |  |
| Smoking status- Never smoker | Ref |  |  |  |  |  |  |  |  | |  |  |  |  | |  |  |  |  |
| Former smoking | -0.099 | -0.14 | -0.06 | 0.00 |  |  |  |  | -0.025 | | -0.06 | 0.01 | 0.20 |  | |  |  |  |  |
| Currently smoking | -0.137 | -0.21 | -0.07 | 0.00 |  |  |  |  | -0.103 | | -0.17 | -0.03 | 0.01 |  | |  |  |  |  |
| Body mass index- Normal | Ref |  |  |  |  |  |  |  |  | |  |  |  |  | |  |  |  |  |
| Overweight | -0.126 | -0.17 | -0.08 | 0.00 |  |  |  |  | -0.060 | | -0.10 | -0.02 | 0.01 |  | |  |  |  |  |
| Obesity | -0.116 | -0.16 | -0.07 | 0.00 |  |  |  |  | -0.030 | | -0.08 | 0.02 | 0.24 |  | |  |  |  |  |
| Diabetes | -0.155 | -0.20 | -0.11 | 0.00 |  |  |  |  | -0.057 | | -0.11 | -0.01 | 0.03 |  | |  |  |  |  |
| Hypertension | -0.161 | -0.20 | -0.12 | 0.00 |  |  |  |  | -0.036 | | -0.08 | 0.00 | 0.08 |  | |  |  |  |  |
| Depression | -0.109 | -0.16 | -0.06 | 0.00 |  |  |  |  | -0.063 | | -0.12 | -0.01 | 0.02 |  | |  |  |  |  |
| Cancer | -0.106 | -0.16 | -0.05 | 0.00 |  |  |  |  | -0.005 | | -0.06 | 0.05 | 0.86 |  | |  |  |  |  |
| Nutritional risk- Not at high nutritional risk | Ref |  |  |  |  |  |  |  |  | |  |  |  |  | |  |  |  |  |
| At high nutritional risk | -0.093 | -0.13 | -0.05 | 0.00 |  |  |  |  | -0.025 | | -0.07 | 0.02 | 0.24 |  | |  |  |  |  |
| Social support- Low | Ref |  |  |  |  |  |  |  |  | |  |  |  |  | |  |  |  |  |
| High support (score ≥4) | 0.117 | 0.08 | 0.16 | 0.00 |  |  |  |  | 0.042 | | 0.00 | 0.09 | 0.07 |  | |  |  |  |  |
| Hearing rate- Excellent, very good, good | Ref |  |  |  |  |  |  |  |  | |  |  |  |  | |  |  |  |  |
| Fair and poor | -0.141 | -0.20 | -0.08 | 0.00 |  |  |  |  | -0.042 | | -0.10 | 0.02 | 0.17 |  | |  |  |  |  |

Appendix F. Regression results of movement activity patterns and cognitive change scores with interaction by employment status

|  | Memory | | | | Executive function | | | | Overall cognition | | | |
| --- | --- | --- | --- | --- | --- | --- | --- | --- | --- | --- | --- | --- |
|  | β | 95CI | | p- value | β | 95CI | | p- value | β | 95CI | | p- value |
| Sedentary/disturbed sleep | Ref |  |  |  | Ref |  |  |  | Ref |  |  |  |
| Interm active-normal short sleep | 0.054 | 0.00 | 0.11 | 0.04 | 0.016 | -0.04 | 0.07 | 0.56 | 0.044 | -0.01 | 0.10 | 0.10 |
| Active-normal sleep | -0.010 | -0.09 | 0.06 | 0.79 | 0.004 | -0.08 | 0.08 | 0.92 | -0.002 | -0.08 | 0.07 | 0.97 |
| Employed | Ref |  |  |  | Ref |  |  |  | Ref |  |  |  |
| Unemployed | -0.061 | -0.12 | 0.00 | 0.05 | -0.075 | -0.14 | -0.01 | 0.02 | -0.084 | -0.14 | -0.02 | 0.01 |
| Class-assignment#Employment |  |  |  |  |  |  |  |  |  |  |  |  |
| Inter-act/normal sleep#Unemployed | 0.015 | -0.06 | 0.09 | 0.71 | 0.073 | -0.01 | 0.15 | 0.08 | 0.050 | -0.03 | 0.13 | 0.21 |
| Active/normal sleep#Unemployed | 0.014 | -0.10 | 0.13 | 0.81 | 0.005 | -0.12 | 0.13 | 0.93 | 0.010 | -0.11 | 0.13 | 0.87 |
| *Fully adjusted models |  |  |  |  |  |  |  |  |  |  |  |  |
|  | Memory | | | | Executive function | | | | Overall cognition | | | |
|  | β | 95CI | | p- value | β | 95CI | | p- value | β | 95CI | | p- value |
| Interm active-normal short sleep | Ref |  |  |  | Ref |  |  |  | Ref |  |  |  |
| Sedentary/disturbed sleep | -0.054 | -0.11 | 0.00 | 0.04 | -0.016 | -0.07 | 0.04 | 0.56 | -0.044 | -0.10 | 0.01 | 0.10 |
| Active-normal sleep | -0.064 | -0.14 | 0.01 | 0.10 | -0.012 | -0.09 | 0.07 | 0.77 | -0.046 | -0.12 | 0.03 | 0.25 |
| Employed | Ref |  |  |  | Ref |  |  |  | Ref |  |  |  |
| Unemployed | -0.046 | -0.12 | 0.02 | 0.20 | -0.002 | -0.08 | 0.07 | 0.96 | -0.034 | -0.11 | 0.04 | 0.35 |
| Class-assignment#Employment |  |  |  |  |  |  |  |  |  |  |  |  |
| Sedentary/disturbed sleep#Unemployed | -0.015 | -0.09 | 0.06 | 0.71 | -0.073 | -0.15 | 0.01 | 0.08 | -0.050 | -0.13 | 0.03 | 0.21 |
| Active/normal sleep#Unemployed | 0.000 | -0.12 | 0.12 | 1.00 | -0.067 | -0.20 | 0.06 | 0.30 | -0.040 | -0.16 | 0.08 | 0.52 |

*Fully adjusted models
